# MtDNA sequence features associated with ‘selfish genomes’ predict tissue-specific segregation and reversion

**DOI:** 10.1093/nar/gkaa622

**Published:** 2020-07-27

**Authors:** Ellen C Røyrvik, Iain G Johnston

**Affiliations:** Department of Clinical Science, University of Bergen, Norway; K.G. Jebsen Center for Autoimmune Diseases, University of Bergen, Norway; Department of Mathematics, University of Bergen, Norway; Alan Turing Institute, London, UK

## Abstract

Mitochondrial DNA (mtDNA) encodes cellular machinery vital for cell and organism survival. Mutations, genetic manipulation, and gene therapies may produce cells where different types of mtDNA coexist in admixed populations. In these admixtures, one mtDNA type is often observed to proliferate over another, with different types dominating in different tissues. This ‘segregation bias’ is a long-standing biological mystery that may pose challenges to modern mtDNA disease therapies, leading to substantial recent attention in biological and medical circles. Here, we show how an mtDNA sequence’s balance between replication and transcription, corresponding to molecular ‘selfishness’, in conjunction with cellular selection, can potentially modulate segregation bias. We combine a new replication-transcription-selection (RTS) model with a meta-analysis of existing data to show that this simple theory predicts complex tissue-specific patterns of segregation in mouse experiments, and reversion in human stem cells. We propose the stability of G-quadruplexes in the mtDNA control region, influencing the balance between transcription and replication primer formation, as a potential molecular mechanism governing this balance. Linking mtDNA sequence features, through this molecular mechanism, to cellular population dynamics, we use sequence data to obtain and verify the sequence-specific predictions from this hypothesis on segregation behaviour in mouse and human mtDNA.

## INTRODUCTION

Mitochondria are metabolically central organelles contained in almost all eukaryotic cells. Due to their evolutionary history, mitochondria retain genomes (mtDNA) which encode machinery important for mitochondrial functionality. There may be hundreds or thousands of copies of mtDNA in a given cell, forming a dynamic population of individual molecules which replicate and degrade quasi-independently of the cell cycle (1).

As a result of mutation, experimental manipulation, or gene therapies, mtDNA populations within cells may consist of a mixture of different mtDNA sequences co-existing. This situation is known as heteroplasmy (2,3). In heteroplasmic mixtures, segregation bias—the systematic profileration of one haplotype over another over time—is commonly observed (1–2,4). For example, a cell starting with a mixture of 50% haplotype A and 50% haplotype B may after one year retain 80% haplotype B and only 20% haplotype A. This segregation is of pronounced medical importance as it can lead to the amplification of a disease-causing mutation if present on the proliferating haplotype (4). However, the causes of segregation bias remain largely unknown, challenging our basic biological understanding and our ability to optimally plan gene therapies.

Most systematic work on segregation bias has been performed in mouse models (5–13), where pairs of haplotypes in a heteroplasmic context are seen to exhibit complex but consistent tissue-specific segregation behaviour (Table 1). For example, segregation bias is often observed to favour one haplotype in liver and a different haplotype in blood (with this behaviour observed consistently across different hematopoeitic cell populations (9)). Tissue-specific directions of segregation bias appear consistent across different nuclear backgrounds (8,9) (although the magnitudes of segregation bias may vary according to nuclear context, with several nuclear loci implicated (9,14–15)). Segregation bias has also been observed in *Drosophila* (16,17) and livestock (18,19) models, including recent work in minipigs (20), and with tunable levels of mtDNA selection in *Drosophila* (21). Recently, human stem cell assays have also shown segregation bias (22,23). Here, mirroring gene therapies, heteroplasmic cells are created using a maternal cell from which most mitochondria are removed, and a ‘donor’ cell providing a replacement supply of mitochondria. In many maternal-donor pairings, the donor mtDNA remains stable, but some pairings exhibit ‘reversion’: segregation bias that favours the initially small amount of maternal mtDNA and amplifies the maternal type to dominate the cell. This reversion presents a potential issue for the recently UK-approved mitochondrial replacement therapies, where technical limitations entail the creation of heteroplasmy with a minority presence of pathogenic mtDNA types that could come to outcompete non-pathogenic molecules (4).

**Table 1.** Segregation bias in mouse models. Nuclear context and haplotype pairs for which tissue-specific segregation bias is observed in mouse models, and the direction (or absence) of that bias. ^1^ Significance patterns of observed segregation are ambiguous due to presentation. ^2^ Segregation patterns are ambiguous due to absence of reference tissue, but liver and brain segregate significantly differently

Reference(s)	Nuclear context	mtDNA type 1	mtDNA type 2	Type 1 increase	Type 2 increase	Neither/ambiguous
5,9–10)	(various, diverse)	NZB	BALB	Liver, kidney	Blood, spleen	Brain, muscle, lung, tail, heart
(8)	RR × DBA or C57BL/6	RR	C57BL/6	Liver, kidney, ovary, stomach, gut (relative to lung^1^)	Brain, heart (relative to lung^1^)	Spleen? (relative to lung^1^)
(11)	JF1 × 129	JF1	C57BL/6	Liver? (relative to tail^2^)	Brain? (relative to tail^2^)	Kidney, tail^2^?
(7)	C57BL/6	NZB	129S6	Liver, kidney, tail, brain	Skeletal muscle, seminal vesicle, ovary, pancreas, spleen	Lung, heart
(12)	C57BL/6	LE	C57BL/6	Gut, spleen, liver, kidney, lung	-	Testis, tail, skin, uterus, blood, heart, muscle, brain
(12)	C57BL/6	HB	C57BL/6	Blood, spleen, liver, lung	Heart, muscle	Gut, testis, tail, skin, uterus, kidney, brain
(12)	C57BL/6	BG	C57BL/6	Gut, testis, tail, skin, uterus, blood, spleen	-	Liver, kidney, lung, heart, muscle, brain
(12)	C57BL/6	ST	C57BL/6	Gut, testis, tail, skin, uterus, blood, spleen, liver, kidney, lung, muscle	-	Heart, brain
(13)	NZW	NZW	C57BL/6	-	-	Brain, heart, lung, liver, kidney, stomach, gut, spleen, muscle, adipose, skeleton, bladder, gonads, brain, skin

The basis for this segregation bias remains poorly understood, despite substantial recent discussion motivated by this importance for gene therapies (22,24–25). Some nuclear-encoded genes influence somatic segregation bias (9,14–15). Some evidence exists for segregation bias being more common and stronger in pairings of more genetically diverse haplotypes, suggesting that mtDNA sequence differences play at least some mechanistic role (12,13). However, further observations suggest that genetic distance alone is not sufficient to predict segregation bias (23). It is therefore likely that differences at specific regions play a role, and overall genetic distance just increases the probability of differences at these specific regions.

Fundamentally, the proliferation of a particular mtDNA type in a tissue depends both on the replication of individual mtDNA molecules and the survival of the organelles and cells that contain them. This survival relies on the expression of respiratory machinery from mtDNA. MtDNA has a unique ‘replication-transcription switch’ (26): unlike the situation in the nucleus, replication of mitochondrial DNA is wholly and directly dependent on transcription, by the single, standard mitochondrial RNA polymerase, POLRMT. POLRMT initiates transcription at the light strand promoter (LSP), and will either continue, producing a full polycistronic transcript, or be attenuated, forming an RNA primer that the replicative mitochondrial DNA polymerase (pol γ) can use to produce another copy of the mitochondrial genome (27).

Many factors affect mtDNA replication and mitochondrial fitness and survival. Cell-wide factors including TFAM and POLRMT (28,29) and bioenergetic and metabolic demands affect the levels of replication and transcription throughout the whole mtDNA population. Mutations in the coding regions of mtDNA may lead to a subset of molecules within the cell producing dysfunctional or toxic gene products; subsequent quality control of mitochondria can clear these pathological mutations (30). Each of these whole-cell and sequence-specific factors can influence mtDNA dynamics and cell fitness.

Here, we consider a different class of mtDNA feature proposed to act in concert with these whole-cell and within-cell factors: sequence features that do not directly influence the functionality of gene products, but affect the balance of replication and transcription for a given mtDNA molecule. This balance of replication and transcription contributes to the *selfishness* of a given mitochondrial genome (31,32). Consider a cell with an admixture of two mtDNA types, with negligible differences in the gene products arising from these two types. If one type undergoes replication more readily, and one type more readily undergoes transcription, the first type may be regarded as more ‘selfish’, favouring its own proliferation over the contribution of functional metabolic components to the cell. In this picture, we consider the functionality of gene products of different mtDNA molecules to be equivalent but not necessarily perfect. If one sequence harbours variants that reduce functionality or cause other cellular issues relative to the other, this constitutes an independent axis of selection that will shape mtDNA dynamics in addition to the replication-transcription balance (1).

Specific examples of selfish mtDNA have been known for some time (33,34), including in plants (where mtDNA features can cause male sterility (35,36)), yeast (where ‘petite’ mutants are highly replicative without encoding the usual complement of mitochondrial machinery (37)), and human (where a selfish deletion mutant causes myopathy (38)). Theoretical work has suggested that different levels of organelle- or cell-level selection can influence the balance of selfish and less selfish mtDNA types (36). Elegant experiments have accordingly shown that different balances between selfish drive and purifying selection result in different patterns of mtDNA proliferation in *Drosophila* (21) and yeast (37), and shown the interactions between ‘hitchhiking’ selfish mtDNA and cellular control in nematodes (39).

In this article, we show that this replication-transcription balance coupled with cell-level selection can account for complex observed tissue-dependent mtDNA segregation patterns. Synthesising published *in vitro* biochemical data, we identify specific sequence features, related to G-quadruplex formation, that predict the behaviour of an individual mtDNA haplotype under this model and show that these features predict both tissue-specific mtDNA behaviour in mice and haplotype-specific reversion in human stem cells. Taken together, our results suggest that the stabilities of G-quadruplex forming sections of the mtDNA control region determine sequence ‘selfishness’ and are important contributors to tissue-specific mtDNA segregation bias.

## MATERIALS AND METHODS

### Mouse data

We use observations from (5,7,8,11-13) and report those tissues where statistically significant segregation bias was reported. Where statistical calculations were absent, we report tissues where segregation bias relative to a reference tissue (typically lung, generally observed to show weak if any segregation bias) was consistently observed.

### Human stem cell data

The correction (40) to human mtDNA data in (22) was used to reconstruct human mtDNA sequences, which are given for reference in Supplementary Information. The labels in Figure 4 refer to specific pairings from (40), with haplotypes: (1) U5a/H1b; (2) U5a/H1b; (3) B2k/H49; (4) X2c/D4a; (5) H56/H2a; (6) H2a/H56; (7) H2a/H56; (8) H44a/H13a; (9) H1b/U5a; (10) H1b/U5a; (11) U5a/H1g; (12) H1g/U5a; (13) h1e/D1f; (14) h1e/D1f; (15) h1e/D1f; (16) D4a/A2g; (17) A2g/D4a; (18) T2b/T2; (19) B2k/H49; (20) H1b/H56; (21) H1b/H56; (22) H1b/H56; (23) H1b/H56; (24) F1a/D4a; (25) F1a/H1b; (26) X2c/U5a.

### Human haplotype data

The set of human mtDNA sequences available from NCBI was downloaded and custom code was used to extract the G-quadruplex regions in CSB2 and the TAS-proximal area and the haplotype information included in the accessions (directly available for 6630 records). The full list of accessions and structures is contained in Supplementary Information.

### Other animal data

Ref. (18) has a bovine *Bos indicus* nuclear background, *B. taurus* mtDNA and *B. indicus* mtDNA. The *B. taurus* type is increased through fetal development. Ref. (19) has a porcine Meishan nuclear background, Meishan mtDNA and Landrace mtDNA. Compared to ear, Meishan mtDNA was higher in liver and lower in spleen and blood, although the statistical significance of these effects is not clear.

Accessions used are as follows. *Mouse haplotypes*. JF1 (KR020498.1); NZB/B1NJ (L07095.1); C57BL/6J (AY172335.1); C57BL/6N (KR020497.1); BALB/cByJ (EF108333.1); RR D-loop from Ref. (8) (AB025348.1); C57BL/6 D-loop from Ref. (8) (AB033825.1); LE (KC663618.1); BG (KC663619.1); HB (KC663620.1); ST (KC663621.1); NZW (EF108341.1); 129S6 (differences from L07095.1 listed in SI of (7)). *Pig haplotypes*. Meishan (KM998967.1); Landrace (NC_000845.1). *Cattle haplotypes*. *Bos taurus* (NC_006853.1); *Bos indicus* (NC_005971.1). We performed all alignments with Clustal Omega (41). Alignments of cattle and pig are shown in Supplementary Figure S7; mouse and human stem cell alignments are available online (see Data and Code Availability).

### Modelling mtDNA agents and cell populations

Our modelling framework represents mitochondria as individual agents with a genetic identity and a complement of respiratory proteins, subjected (or not) to selective pressure based on these protein complements. We model a cell as containing *m* mtDNAs, each of which may be of type 1 (with selfishness λ_1_) or type 2 (with selfishness λ_2_). A parameter m* dictates the maximum mtDNA content of a cell; we set m* = 1000 but verified that changes to this parameter did not strongly influence model behaviour. Each mtDNA *i* exists in a mitochondrial element, which also contains an amount *p*_*i*_ of respiratory protein complexes (we will later allow mixing of content between these elements to capture mitochondrial networks and dynamics, see below). Each mtDNA experiences polymerisation initiation events which are random (Poissonian) and occur with rate γ(1 − *m*/m*). An event for mitochondrion *i* leads to replication with probability λ_*i*_ (we will later reduce this probability to capture the probability of replication failure, see below), and transcription with probability (1 − λ_*i*_). If mtDNA *i* replicates, another mtDNA of the same type is added to the population, and it and mtDNA *i* are both assigned *p*_*i*_/2 proteins. If mtDNA *i* undergoes transcription, *p*_*i*_ is increased by 1.

Each mtDNA experiences random protein degradation events with rate κ_*p*_, where *p*_*i*_ is decreased by 1, and random potential mtDNA degradation events with rate κ_*m*_. In this event, mtDNA *i* is removed from the population if *p*_*i*_ < *P*. If *p*_*i*_ ≥ *P*, nothing happens for this event. *P* is thus a threshold amount of respiratory machinery required for adequate mitochondrial function.

We fix λ_1_ and consider the balance of haplotypes over time with different λ_2_ and *P*. We simulate the system for 1000 timesteps (enough for transient behaviour to disappear and a consistent readout of selective shifts to emerge, Supplementary Figure S2) for γ = κ_*m*_ = 0.1, κ_*p*_ = 0.01 (different values do not qualitatively change our results). Supplementary Figure S2 shows typical time behaviours of copy number, heteroplasmy and protein statistics over time in the simulation. Figure 2 uses λ_1_ = 0.3; consistent behaviour is observed for different λ_1_ (Supplementary Figure S1).

In the Supplementary Information we describe adaptations of this model to consider cell-level rather than organelle-level selection and show the same behaviour under several model structures and parameterisations. We also consider structural variations of the core model. First, we model the possibility of the failure of replication initiation with a parameter *ρ* describing the probability that, when the decision is made to replicate an mtDNA molecule, the replication event fails to initialise and so no change is made to the population (Supplementary Figure S3). This noise partially decouples effective replication rate from the current state of the system *m*. Second, we model the sharing of protein content between mitochondrial elements with a diffusion-like term (Supplementary Figure S4). Specifically, *p*_*i*_ = *D*(〈*p*〉 − *p*_*i*_), where 〈*p*〉 is the mean protein content across all mitochondrial elements. High *D* homogenises the population; *D* = 0 keeps the protein complements associated with different mtDNA molecules entirely separate. *D* > 0 thus accounts for a ‘leaky’ genotype-phenotype relationship between an mtDNA molecule and its protein products, which may arise through exchange of mitochondrial content via mitochondrial dynamics and network structure (42,43).

### Statistical methods

For model comparison, we used Bayesian logistic regression with the response variable ‘did reversion occur?’ and predictor variables describing the differences in guanine counts in CSB2 and the three TAS regions. This Bayesian regression was implemented using the *arm* package in R (44), using an approximate EM algorithm and Student-t prior distributions for regression coefficients, as numerical issues occurred when fitting some model incarnations using built-in generalized linear model functionality. The Bayesian Information Criterion was used to select the best model; the corrected Akaike Information Criterion gave comparable results although suggested a role for TAS_G4a as well as TAS_G4c (Supplementary Table S1). Predictive performance was calculated by holding back half the non-reverting and half the reverting observations as a test set, training the logistic model on the remaining data, then assessing its predictions on the test set. This procedure was performed 10^3^ times with different samples of half the observations in each case. Analysis was performed in R (45); McFadden’s pseudo-*R*^2^ value was computed using the *pscl* package (46).

## RESULTS

### A replication-transcription-selection model predicts saddle-like patterns of mtDNA segregation bias

**Figure 1.**
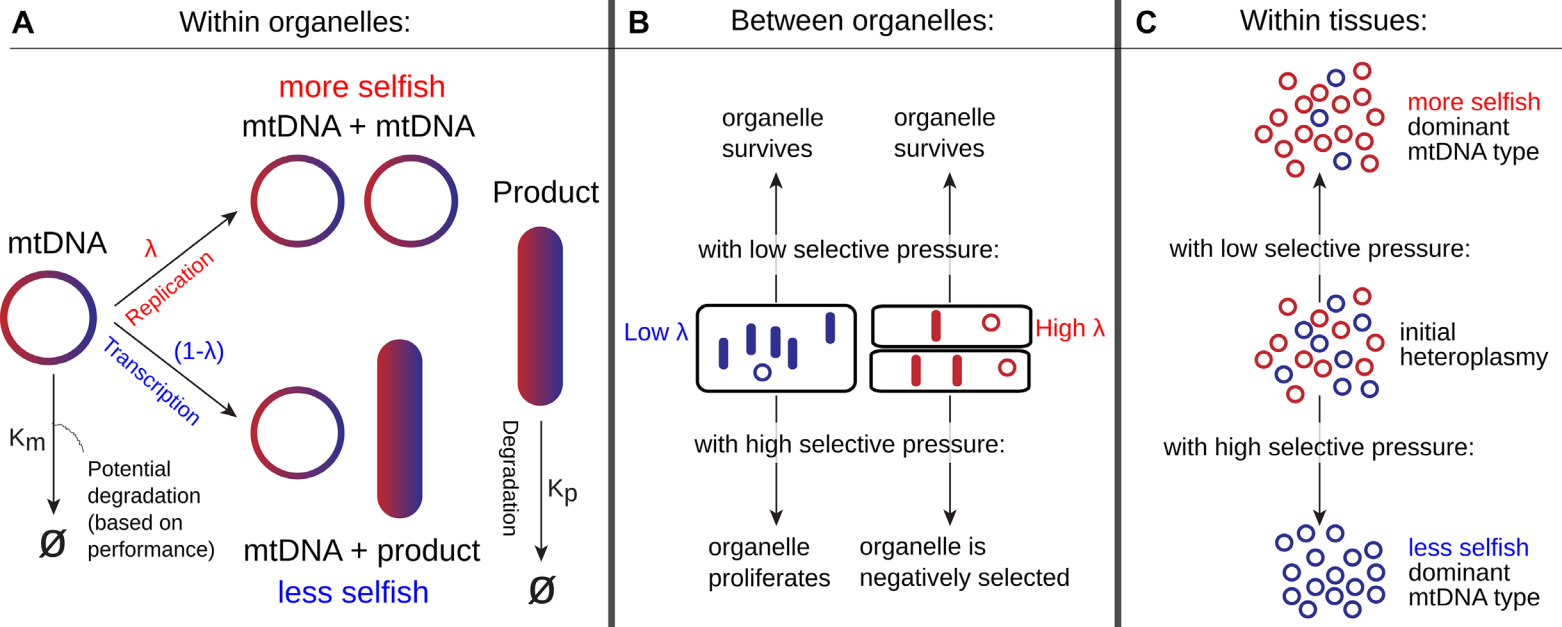
The replication-transcription-selection model predicts different segregation bias in different tissues. Individual mtDNA molecules may undergo selfish replication (with rate λ) or co-operative transcription and gene expression (with rate (1 − λ)). Cells containing many selfish mtDNAs will quickly accumulate more selfish mtDNA, but will lack respiratory machinery; cells containing less selfish mtDNAs will less quickly accumulate less selfish mtDNA, but will have higher levels of respiratory machinery. If there is selective pressure for respiratory capacity (most manifest in tissues with high cell turnover), cells dominated by selfish mtDNAs will be removed in favour of those with less selfish mtDNAs that have maintained cellular performance. Where there is little selective pressure (or little cell turnover to manifest such pressure), the selfish mtDNAs will come to dominate the tissue due to their unchecked replicative advantage.

We first sought to construct a general theory with which to understand and predict tissue-specific patterns of mtDNA segregation. Following the above idea of the replication-transcription switch (26), we assume that some sequence features of a given mtDNA molecule (to be determined later) determine that molecule’s balance between replication and transcription. MtDNA molecules favouring replication are in a sense more ‘selfish’, more readily undergoing replication and less readily producing respiratory machinery of use to the cell. MtDNA molecules favouring transcription are correspondingly less ‘selfish’. Over time, each molecule undergoes replication and transcription events, respectively producing more mtDNA (of no direct value to the cell) and polycistronic transcripts (which can be translated and contribute to the cell’s bioenergetic poise) according to its particular balance (Figure 1A).

We reiterate that many other factors will influence the transcriptional activity of a given mtDNA in a given tissue, including signalling and nuclear control (for example, via TFAM expression (28)), levels of polymerase (29), metabolic poise, and others. However, our theory focusses purely on the *relative* difference in activity between mtDNA types, and its predictions hold under variability in these overall levels (see below).

We then consider a situation where two mtDNA types, with different replication-transcription balances, co-exist in the same cell. We then have a more selfish mtDNA type favouring replication co-existing with a less selfish mtDNA type favouring transcription. In the absence of any other effects, the more selfish type will replicate more quickly, and over time come to genetically dominate over the less selfish type (Figure 1B and C).

However, the bioenergetic capacity of the mtDNA’s ‘host’ mitochondria must also be considered. Selfish mtDNA types will produce less respiratory machinery and contribute less to bioenergetic performance. Organelles containing selfish mtDNA types may then be expected to experience more functional challenges than those containing less selfish mtDNA types, which produce more machinery and contribute more to performance. If organelles are selected for bioenergetic capacity, as is known to occur through mitophagy and recycling (30), those mitochondria containing less selfish mtDNAs will then preferentially survive, while those less functional mitochondria containing more selfish mtDNA will be recycled. This selection acting at the organelle and/or cellular level can then balance, or reverse, a replicative advantage at the molecular level, as found in quantitative models of plant mtDNA populations (36) and in models and experiments on yeast (37) and *Drosophila* (21).

It is thus easy to see how selfishness can be either a successful or unsuccessful strategy depending on the level of selection for bioenergetic capacity that acts within cells (Figure 1B and C). In tissues with low mitochondrial and/or cellular turnover, and/or low energetic requirements, nothing prevents selfish mtDNAs proliferating and dominating the tissue. By contrast, when higher organelle and/or cellular turnover allows mitochondria to be selected based on bioenergetic performance, unselfish mtDNAs will come to dominate (as selfish mtDNAs compromise the performance of their host organelle).

We underline that we are suggesting a mechanism that describes the *relative difference* in replication and transcription between two mtDNA types in the same cell. TFAM, POLRMT, bioenergetic and metabolic poise, and many other dynamic influences affect the *global* amount and balance of replication and transcription across mtDNAs (28,29). Our theory describes the *relative* difference in behaviour of two mtDNA sequences on this given global background. If, for example, global rates of mtDNA replication are limited, the relative replicative poise of each mtDNA type can remain intact, so that the overall patterns of relative proliferation remain unchanged. Further, we underline that our suggested mechanism acts *in addition to* selection arising from functional or other differences between mtDNA gene products. Here we assume that mtDNA sequences differ only in their replication-transcription balance, with no fitness differences due to gene product function or production. Any such differences will constitute another axis of organellar selection (see Discussion).

To translate this theoretical picture into a quantitative model, we assign each mtDNA type a parameter λ describing its replication-transcription balance, or, equivalently, its ‘selfishness’. Replication or transcriptional events take place at random, following the ‘relaxed replication’ paradigm (47) that has been employed in related recent studies on mtDNA dynamics (48). Each time an event occurs, λ gives the probability that the mtDNA molecule will replicate; (1 − λ) is correspondingly the probability that it will follow the transcription route.

Several previous studies have considered the dynamics of mixed mtDNA populations with functional differences between types (49). However, these studies do not typically consider expression of respiratory machinery (50–53), although it can be pictured as a general mitochondrial quality state (37,54). To explicitly capture the transcription-replication balance, we consider each mtDNA to be contained within a mitochondrial element. Transcription increases the amount of respiratory machinery in this element; replication induces a division into two elements, each inheriting half the previous element’s complement of respiratory machinery. Mitochondrial elements are randomly marked for degradation, and degradation proceeds if a marked element possesses less than a given threshold amount of respiratory machinery.

**Figure 2.**
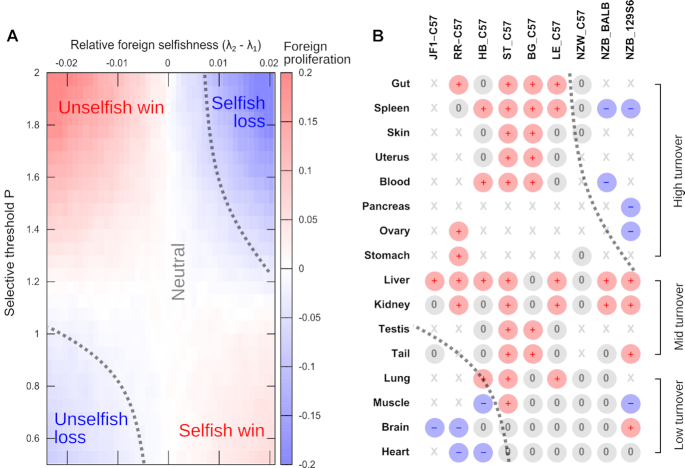
The RTS model predicts the saddle-like tissue-dependent segregation bias observed in mouse models. (**A**) Segregation bias under the RTS model, as a function of the selfishness difference (λ_2_ − λ_1_) between mtDNA types (horizontal axis) and the strength of organelle-level selection, manifest as the threshold value of respiratory protein content *P* required for organelle survival (vertical axis). At low levels of selection, the more selfish mtDNA type proliferates; at higher levels of selection, less selfish mtDNAs are favoured due to their improved contribution to bioenergetic performance. (**B**) Observed patterns of segregation in mouse mtDNA pairings from Table 1. Tissue-specific behaviour follows the predictions of the RTS model and the resulting ordering of haplotype pairs provides an estimate of relative selfishness for each pairing.

As shown in Figure 2A and Supplementary Figure S1, this simple model predicts that the magnitude and direction of segregation bias will depend on the selfishness difference between mtDNA types and the level of selection acting at the organelle level. Specifically, ‘saddle’-like behaviour is predicted, where an mtDNA type will be favoured if it is highly selfish in low-turnover tissue, or if it is less selfish in high-turnover tissues. When little selection acts, selfish mtDNA types can replicate without consequence and come to dominate the cell. By contrast, when selection for bioenergetic capacity is imposed, organelles (or cells) containing selfish mtDNAs are functionally challenged and therefore removed, leading to an overall accumulation of less selfish mtDNAs. The overall picture involves the direction of segregation bias changing (from favouring selfish to favouring less selfish mtDNAs) as selection increases, producing a saddle-like structure (Figure 2A).

This saddle-like behaviour is predicted robustly under different structures and parameterisations of the RTS model (Supplementary Figure S2). In particular, it is only the *relative* rate of transcription versus replication (given by λ) for a given mtDNA molecule that influences the direction of segregation bias; the *absolute* rates just influence the timescale at which this bias becomes manifest. The structure of the saddle-like behaviour remained unchanged when we introduced noise in replication initiation (Supplementary Figure S3) and partial homogenisation of protein content between mitochondrial elements (Supplementary Figure S4), with the latter representing the case where protein content is shared through a mitochondrial network or through transient fusion events (42).

The results in Figure 2A show the mean behaviour of haplotypes, but deviations from this mean behaviour are to be expected due to intrinsic cellular noise. Under the RTS model, cell-to-cell variability in mtDNA proportions increases linearly with time, following predictions of (48) and observations of (55) (Supplementary Figure S2). We also consider a variant of this model where selection instead acts at the cellular level and show that the results are qualitatively identical (Supplementary Figure S5). These general observations agree with previous studies investigating the action of organelle- or cell-level selection on differently-replicating molecules in the context of plant mtDNA (36).

### The RTS model accounts for the complex patterns of tissue-specific segregation in mouse models

We next asked whether our model could account for the tissue-specific patterns of segregation bias observed in mouse models (Table 1). These data together display some structuring across tissues and mtDNA pairings. If haplotypes are classified according to which proliferates in liver (the most consistently observed segregation), three classes of behaviour are broadly observed across tissues. Some (kidney, testis) consistently follow the liver segregation pattern regardless of haplotype pairing. Others (spleen, blood, pancreas) show a different direction to liver in pairings involving NZB, and follow liver in other pairings. Still other tissues (muscle, brain, heart) show a different direction to liver in pairings involving JF1, RR and HB, and are comparatively neutral otherwise.

These tissue classes are broadly separable by cellular, and mtDNA, turnover rates, as found in (12). Pursuing this link, we ranked tissues by their estimated selectivity for bioenergetic capacity, a combination of cell turnover (allowing selection) and bioenergetic demand (imposing selection) – see Methods. We then found that the predicted saddle structure from the RTS model was reflected in the observed mouse data (Figure 2B). We assigned haplotype pairs to one of three classes (mtDNA 1 more selfish, similar to, or less selfish than mtDNA type 2) and found that 74% (37/50) of nonzero segregation bias observations were accounted for by this classification. The overall classification success rate (0.66, 61/92) was significantly better than random assignment (*P* = 2.5 × 10^−5^, binomial test with *B*(92, 0.504) reflecting the proportion of nonzero observations). This fit to observed data predicts relative selfishness differences between pairs of observed haplotypes (i.e., the ordering on the horizontal axis of Figure 2B): (JF1, RR, HB, ST) < C57; (BG, LE, NZW) ≃ C57; (BALB, 129S6) < NZB.

### G-quadruplexes in the mtDNA control region influence replication-transcription balance

**Figure 3.**
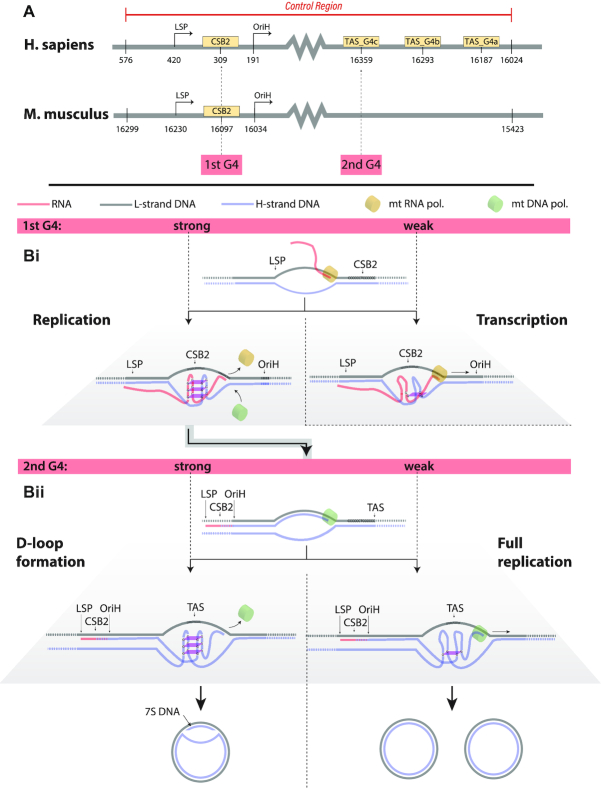
Control region features and hypothesised association with ‘selfishness’. (**A**) Selected positions in the human and mouse mtDNA control regions that possess sequence motifs expected to facilitate hybrid G-quadruplex formation. (**B**) G-quadruplex influence on replication-transcription balance. (i) After transcription initiation, a strong G-quadruplex at CSB2 may lead to RNA polymerase stalling and termination of transcription, while a weaker G-quadruplex allows transcription to proceed. Aborted transcription leads to primer formation, initiating mtDNA replication. (ii) After replication is initiated, further strong G-quadruplex regions may similarly interfere with DNA polymerase progress, causing D-loop formation rather than full replication. A single, strong G-quadruplex at CSB2 may thus be viewed as the strongest facilitator of replication.

We next asked what specific sequence features might be associated with λ, the selfishness of a given mtDNA molecule, and why. In particular, we considered sequence features that may affect the balance of the replication-transcription switch mentioned above (26). Recent discussion has focussed on a particular section of the mtDNA control region called CSB2 (conserved sequence box II) (22,24–25) (Figure 3A), which has long been suggested to regulate the rate of human mtDNA synthesis (56).

A feature of CSB2 is its potential to form a hybrid G-quadruplex structure involving the complementary mtDNA strand and its nascent RNA transcript (56). G-quadruplexes are DNA secondary structures consisting of stacked planar guanine tetrads that play a number of regulatory roles, including in nuclear DNA replication, telomeres and transcription in a range of metazoans (57,58). CSB2 typically involves a series of cytosine bases broken by a central feature: *C*_6 − 9_*N*_1 − 3_*C*_5 − 6_, with the corresponding guanine bases in the complementary strand and nascent transcript capable of forming a quadruplex structure. In mice, for example, the central feature usually consists of one or two adenine bases.

Previous studies have noted that differences in the number of guanine bases in CSB2 are associated with segregation bias (reversion) between mtDNA sequences in human stem cells (22,24–25). We hypothesise, cf. (56), that the stability of the associated G-quadruplex structure plays a role in determining the replication-transcription balance (selfishness) for a particular mtDNA sequence (Figure 3B).

*In vitro* experiments exploring transcription termination for a large variety of CSB2 structures found that termination occurred more readily for regions with more guanine bases (59). Independent *in vitro* mtDNA assays showed that shorter guanine tracts resulted in fourfold decreased replication primer formation (22). Biochemical observations confirm that G-quadruplexes formed with more guanine residues are more thermodynamically stable (60), and sequence-independent investigation shows that smaller nucleotide loops between guanine residues also stabilise the G-quadruplex (61). Taken together, these observations suggest a picture where more guanine residues, more tightly associated, produce stronger G-quadruplex structures (62), which more readily stall transcription (59) and thus more readily yield replication primers (22) (Figure 3B). This stalling may result from physical factors including tension in the elongating strand, or torsion or conformational changes induced by the hybrid G-quadruplex, either of which effects would be more potent with a ‘stronger’, less easily resolvable G-quadruplex. Hence, sequence features that favour transcription termination naturally make an mtDNA molecule more ‘selfish’: both limiting gene expression and promoting replication (Figure 3B).

CSB2 has received the most attention in the literature, but our hypothesized mechanism also predicts a potential role for other G-quadruplex forming mtDNA regions. Once a primer has formed after stalling at CSB2, and DNA replication has begun, any further unresolved stalling elsewhere will disfavour replication (Figure 3B). We therefore predict that a strong G-quadruplex at CSB2 will increase selfishness, but that strong G-quadruplexes elsewhere will decrease selfishness by challenging DNA replication. In particular, C_3+_N_1−7_C_3+_ potential intermolecular G-quadruplex forming motifs are found near the termination associated sequence (TAS) region in mtDNA also (see below). Our model predicts that weaker G-quadruplexes in this region will increase selfishness.

### G-quadruplex sequences in the control region predict segregation patterns in mice and human stem cells

**Figure 4.**
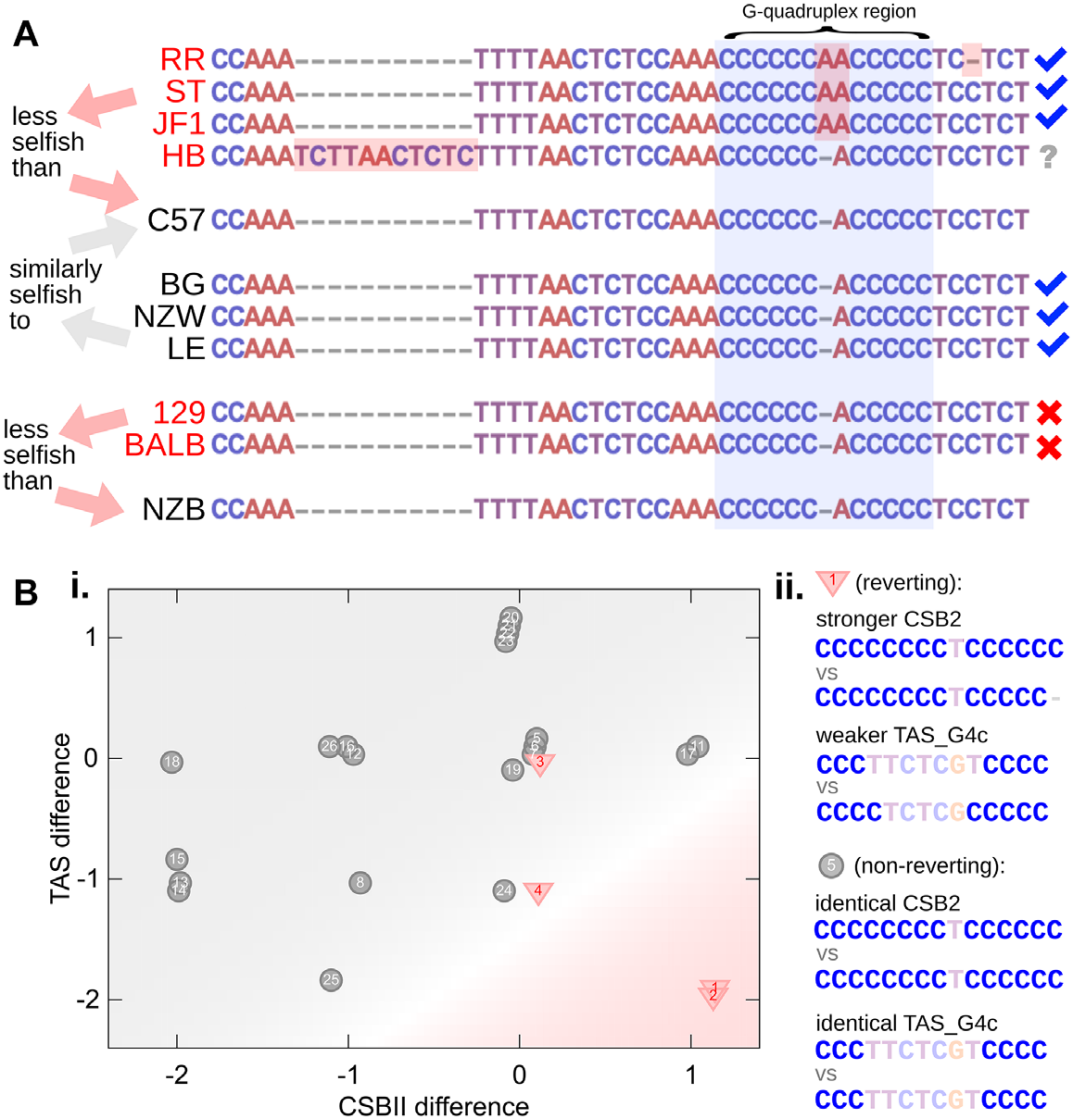
G-quadruplex features predict segregation patterns in mouse and human. (**A**) CSB2 and surrounding loci in mouse haplotypes from Table 1. Predicted selfishness relationships from fitting tissue-specific observations to the RTS model are given on the left. Candidate features modulating G-quadruplex stability are highlighted in red. Ticks, crosses, and question mark denote whether fitted selfishness is predicted by CSB2 region features. (**B**) i. Sequence features in pairs of mtDNA in human cell lines which are observed to show reversion via segregation bias (triangles) or not (circles). Labels denote specific pairings (see Methods). Horizontal axis gives difference in guanine count in CSB2 G-quadruplex region; vertical axes gives difference in guanine count in TAS G-quadruplex region. Shaded background shows the best-fit logistic regression model (see text) predicting reversion (pink) or no reversion (grey). ii. Example potential G-quadruplex sequences in a reverting and non-reverting case.

To investigate evidence pertaining to our hypothesised mechanism, we first analysed the mtDNA sequences of mouse haplotypes from the pairings in Figure 2B. Alignment of the mouse mtDNA haplotypes with Clustal Omega (41) found diversity in CSB2 but not in any other potential G-quadruplex forming parts of the control region (Figure 3A). The fit to our RTS model predicted selfishness relationships (JF1, RR, HB, ST) < C57; (BG, LE, NZW) ≃ C57; (BALB, 129S6) < NZB. Correspondingly, relative to C57, JF1, RR and ST all have a destabilising additional adenine base in the centre of their CSB2 G-quadruplex region (RR has an additional proximal deletion of a cytosine base), and HB has a large insertion proximal to CSB2 that may influence G-quadruplex formation (Figure 4A). BG, LE, and NZW had no changes in the CSB2 region relative to C57. We thus found that 6/9 of fitted selfishness differences could be explained by sequence features decreasing the stability of the CSB2 G-quadruplex (59–61) (7/9 if the HB insertion is included; Figure 4A). We were unable to find predictive CSB2 differences between BALB, 129S6, and NZB, but in the particular case of NZB, the previously characterised functional difference between NZB and BALB may provide an alternative target for higher-level selection (63).

To further test our hypothesised link between G-quadruplex stability and selfishness, we analysed data from recent experiments in human embryonic stem cells (hESC) where segregation bias is observed. We reconstructed and aligned the haplotypes involved (see Methods), finding sequence diversity in both in the CSB2 and other potential G-quadruplex forming parts of the control region, specifically in the TAS regions, which we label TAS_G4a-c (Figure 3A). Refs. (22,24,25) have noted that a higher number of CSB2 guanine bases in these pairings is associated with increased propensity for segregation bias. Following our proposed mechanism, we asked whether taking the TAS region into account can improve the predictions of reversion. Of course, an absence of observed reversion in these experiments does not necessarily imply no segregation bias, because the starting amounts of maternal mtDNA may be small enough for random drift to remove even in the absence of a selective advantage. Further, the time scale of hESC culture is likely too short for the sort of tissue level segregation observed in mice to occur.

We used logistic regression with the number of guanine bases in CSB2 and the potential G-quadruplex regions in TAS (Figure 3A) as predictors of reversion. The model including CSB2 and TAS_G4c regions had the lowest Bayesian Information Criterion, suggesting the strongest statistical support (Supplementary Table S1). We found that the CSB2-alone model had a pseudo *R*^2^ value of 0.26, but when TAS_G4c was included this rose to 0.47. The CSB2-TAS_G4c model accounted for 92% (24/26) observed reversion behaviours (nonzero and zero), performing significantly better than random chance (*P* = 0.02, binomial test with *B*(26, 0.74), reflecting the proportion of nonzero observations), and than the CSB2-alone model. When trained on 10^3^ samples of half the observed haplotype pairs from (22) (half the reverting and half the non-reverting pairings), the CSB2-TAS had a mean prediction accuracy of 0.93 for reversion in the independent test pairings (Figure 4Bi). Together, these results support our hypothesis that a strong G-quadruplex in CSB2, and weak G-quadruplexes elsewhere, assign a proliferative advantage to an mtDNA sequence (Figure 4Bii).

Finally, we considered other species where segregation bias has been investigated. These include pig (19) and cattle (18) systems. In pig, some suggestion of tissue-specific segregation bias was observed but we were unable to show that it passed a significance threshold. Correspondingly, we found no diversity in candidate G-quadruplex sections in CSB2 for the haplotypes involved, although a polymorphism in TAS_G4c was present that could potentially lead to smaller selfishness differences (Supplementary Figure S7A). By contrast, in cattle, segregation bias was observed in embryonic development, favouring *Bos taurus* mtDNA over *Bos indicus*, and we identified a sequence difference in a candidate G-quadruplex region predicting a corresponding selfishness difference between mitotypes (Supplementary Figure S7B).

## DISCUSSION

We have proposed a mechanism whereby the replication-transcription balance (‘selfishness’) of an mtDNA molecule can affect its relative proliferation in heteroplasmic cells. The direction and magnitude of this effect in tissues is modulated by cellular selective pressure for bioenergetic capacity, which depends on cell turnover and bioenergetic demand. More selfish genomes proliferate in the absence of cell-level pressure; less selfish genomes, which support cellular function, proliferate when cell-level pressure is higher. Together, these effects can explain much of the diverse tissue-specific behaviour observed in mouse models.

We hypothesise, following biochemical arguments and observations in stem cells, that sequence features affecting the stability of G-quadruplexes in the mtDNA control region play a role in determining the replication-transcription balance of a given mtDNA sequence. These features—particularly, the number of guanine bases in the CSB2 and TAS regions—predict selfishness in mouse haplotypes and reversion propensity in hESCs.

It is likely that segregation bias depends on multiple sequence features as well as nuclear factors and metabolic context (63). Indeed, several nuclear-encoded genes have been found to influence the dynamics of heteroplasmic populations in somatic tissue (9,14–15) and the germline (64). The organelle- and cell-level selection we model are two nuclear-mediated strategies to mitigate against selfish mtDNA replication (31,32), and as such exist in the broader framing of co-operation and conflict between mtDNA and nuclear genes (recently reviewed in (32,65)).

Our model only suggests that differences in replication-transcription balance may account for some of these observed behaviours. We do not intend to claim that G-quadruplex stability is the only or even the leading factor determining segregation bias, but have shown that several observed features of segregation behaviour can be explained by these features, and that a plausible biophysical mechanism may underlie this predictive power. It is well known that mtDNA variants compromising functionality experience selective pressure (for example, the 3243 mutation in humans (66,67)). When one sequence has variants that directly challenge bioenergetic function or other aspects of cellular fitness, these may be acted upon by selective mechanisms and provide contributions to segregation behaviour in addition to those from our model. However, as we describe above, we expect our general findings (concerning the relative behaviour of two mtDNA types) to remain robust to global changes in cellular behaviour that are not specifically linked to only one mtDNA type. For example, a global reduction in mtDNA transcription (perhaps through limited polymerase expression) will not affect the *relative* behavioural differences between the two mtDNA types from which our predicted behaviour emerges.

Further, other potential G-quadruplex-forming regions also exist throughout human and other mtDNA sequences (62,68); while our hypothesis focusses on the control region, the relative stabilities of these features across diverse genotypes may also play a role in mtDNA population dynamics, proposing further hypotheses for pursuit as more data becomes available. To our knowledge, no software tools yet exist that can make thermodynamic predictions about intermolecular DNA-RNA quadruplex stability (although a predictor for such structures does exist (69)). However, as we have argued above, insights from the more general literature on intramolecular G-quadruplexes (60–62) are informative, entropic arguments suggest than long G-tracts increase stability, and this theory is borne out by mtDNA-specific experimental observations of subsequent replication behaviour (22).

Our results are compatible with experiments in yeast (37) and *Drosophila* (21) exploring the tension between selfish drive and higher-level selection in shaping mtDNA populations. Interestingly, recent work exploring human mtDNA germline selection (70) observed pronounced differences between mother and child allele frequencies of several loci associated with potential G-quadruplex forming parts of the control region. While we did not find polymorphisms affecting the CSB2 G-quadruplex in the set of transmitted features in (70), there are several instances of transmission differences for a polymorphism (T16362C) in the section we label TAS_G4c, and in other sections bearing potential G-quadruplex sequence motifs. These observations are compatible with the idea that stability of control region G-quadruplexes influence relative mtDNA proliferation, and suggest further candidate regions for investigation in addition to those we consider here.

Accordingly, our model makes several testable predictions for future experiments: most generally, that mtDNA sequences with more stable CSB2 G-quadruplexes, and less stable G-quadruplexes elsewhere, will show the tissue-specific segregation behaviour associated with less selfish molecules (proliferation in highly selective tissues, loss in weakly selective tissues).

The question of matching mtDNA sequences (‘haplotype matching’) is frequently discussed in the application of mtDNA gene therapies (71). Haplotype matching is proposed as an attempt to avoid problematic segregation bias in gene therapies by selecting a ‘donor’ mtDNA type for which maternal reversion through segregation bias is unlikely. Our model agrees with recent discussion on this topic (24,25) suggesting that the number of guanine bases in CSB2, rather than general sequence similarity, may be a promising consideration for minimising reversion. We would also suggest consideration of the TAS region that our work indicates may also affect segregation bias. Substantial diversity exists in these regions in human mtDNA accessions in NCBI (Supplementary Figure S6) which partially correlates with overall mtDNA haplotype.

While this manuscript was in press, two exciting related developments appeared in the scientific literature. The first reports an mtDNA sequence feature in transmissible canine cancer that seems likely to confer a replicative advantage: an insertion of two C bases at position 16660, next to an existing C tract in the control region (72). It may be possible that this CC insertion may influence the formation of a hybrid G-quadruplex structure at this site, suggesting a possible link to our proposed mechanism modulating mtDNA replication-transcription balance. The second is the report of precise gene editing in mtDNA (73). This groundbreaking development will allow the design and investigation of synthetic mtDNA sequences to explore the mechanisms of segregation bias on an unprecedented scale, providing a clear future route to testing our hypothesised mechanism.

Our RTS model adds an explicit description of mtDNA gene expression to a core of stochastic models for functional (37,50–54) and genetic (36,47–48,74–77) dynamics of mixed mitochondrial populations. There is a growing intersection between this body of work and the wide literature on multilevel selection on competing replicating agents in an evolutionary context (78,79). Theoretical work on model unicellular organisms has explored the effect of mitochondrial bottlenecks, paternal transmission, and cellular selection (77). Previous work in plants, where mtDNA recombination produces diverse cellular mtDNA admixtures of potentially different selfishnesses, has shown that multilevel intra- or intercellular selection provides a way of combatting selfish proliferation (36). The pronounced role for mitochondrial dynamics in imposing intra-cellular quality control (30), captured by many of these models (50–54,75), suggests that tissue-specific mitochondrial dynamics may play an important role in this multilevel selection and mitigation of selfish proliferation (32,80).

## DATA AVAILABILITY

Simulation code for the organelle- and cell-based RTS models, code for bioinformatic and statistical analysis of data from Ref. (25) and the NCBI, visualisation code, and mouse and stem cells alignments are freely available at https://github.com/StochasticBiology/selfish-mtdna.

## Supplementary Material

gkaa622_Supplemental_File
