# Supplementary material for: MtDNA sequence features associated with ‘selfish genomes’ predict tissue-specific segregation and reversion
Source: Nucleic Acids Res. 2020 Jul 27;48(15):8290–301. doi: 10.1093/nar/gkaa622 (PMC7470939; doi:10.1093/nar/gkaa622)
Supplement: gkaa622_Supplemental_File [file gkaa622_supplemental_file.pdf]

Supplementary Information

Cell-level selection

To explore the robustness of our results with respect to model choice, we adapted the modelling framework from the main text to include selection for bioenergetic performance manifest at the cellular rather than the organelle level. This model has the same basic structure as before: we consider individual cells with m_1 type 1 mtDNAs and m_2 type 2 mtDNAs. Under this model, the rate of a polymerisation event occuring to each mtDNA in a cell is given by $\gamma(1 - (m_1 + m_2)/m^*)$. Given a polymerisation event, each mtDNA replicates with rate λ_i and expresses machinery with rate $(1 - \lambda_i)$ as before. MtDNA molecules are degraded with rate κ_m regardless of type or protein statistics, and proteins are degraded with rate κ_p per mtDNA.

Now, we consider a collection of cells evolving over time. Cells with a total protein count under a threshold P_C die and are replaced by a copy of a randomly drawn cell from the surviving population, modelling a sample from a bulk tissue. Selection thus now acts on the total protein complement produced by a group of mtDNAs. A population of $N_C = 100$ cells is initialised with $m_1 \sim U(0, N_C)$ and $m_2 = N_C - m_1$. The system was allowed to evolve for 100 simulation timesteps. With selection acting at this higher level, saddle-like behaviour very similar to the organelle-level model is obtained. Fig. S5 gives a set of examples for $\kappa_m = 0.01$, $\kappa_p = 0.1, \gamma = 0.1$.

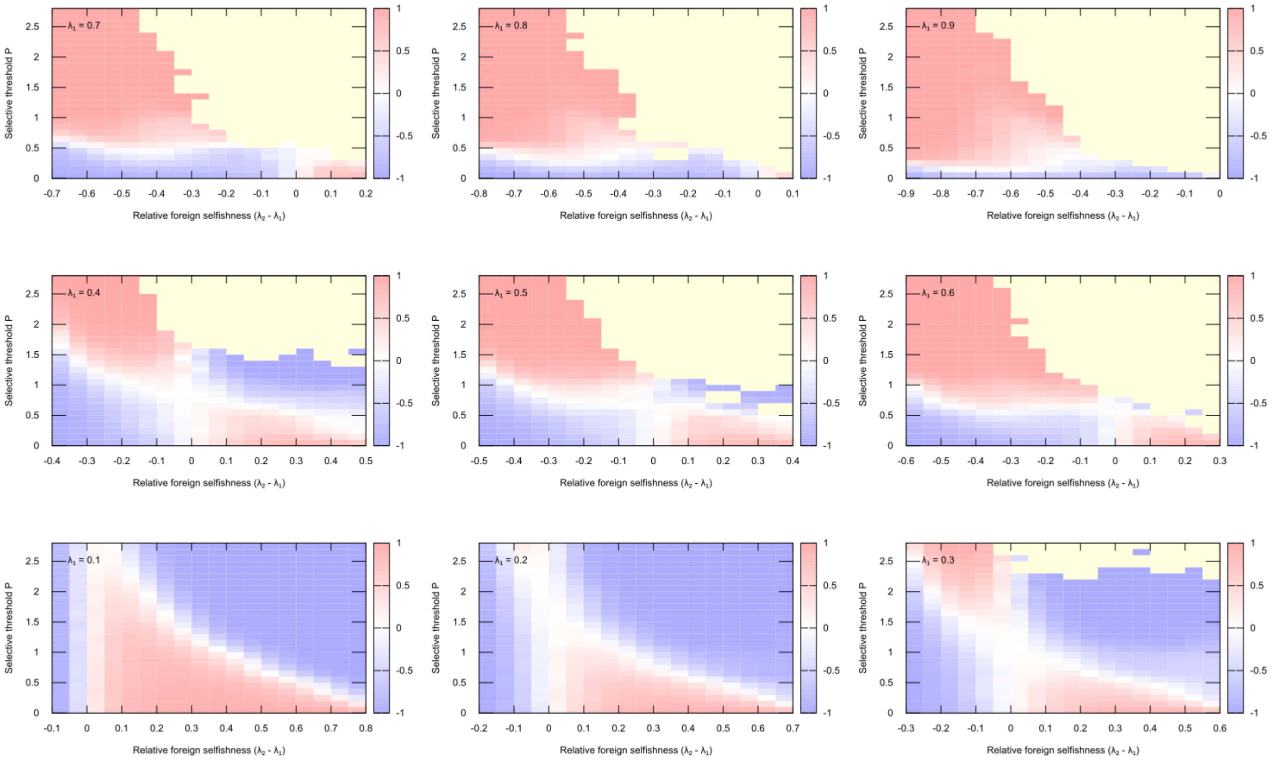

Figure S1: **RTS model under different parameterisations.** Relative proliferation of the 'foreign' mtDNA type is given by colour, as in Fig. 2 in the main text (plotting a region of the $\lambda_1 = 0.3$ case here). Different panels of the trellis correspond to different wildtype proliferation rates λ_1 in the RTS model. Yellow shaded regions (usually at the top right) denote regions of extinction (no mtDNAs are sufficiently unselfish to overcome selective pressure). The saddle-like behaviour seen in experimental observations is preserved across many specific parameterisations.

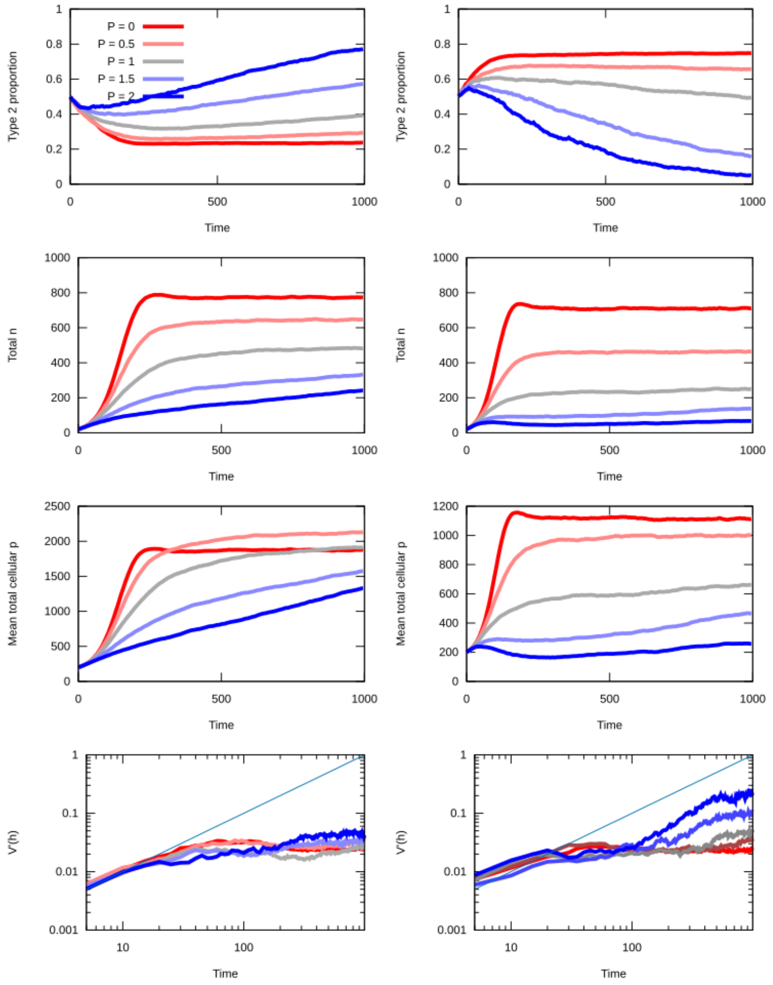

Figure S2: **Time behaviour of RTS model.** The time behaviour of ‘foreign proportion’ (proportion of type 2 mtDNAs), the total number of mtDNAs n, and the heteroplasmy variance $V'(h)$ in the RTS model (organelle level selection) for different parameterisations. (left) $\lambda_1 = 0.4$, $\lambda_2 = 0.2$. (right) $\lambda_1 = 0.4$, $\lambda_2 = 0.7$. Different lines show different selective thresholds P for protein content.

Model	BIC	AICc
CSB2 alone	23.37	21.02
CSB2 + TAS_G4a	26.37	23.12
CSB2 + TAS_G4b	26.56	23.31
**CSB2 + TAS_G4c**	**22.38**	19.13
CSB2 + TAS_G4a + TAS_G4b	29.60	25.67
CSB2 + TAS_G4a + TAS_G4c	22.84	**18.91**
CSB2 + TAS_G4b + TAS_G4c	24.99	21.04
CSB2 + TAS_G4a + TAS_G4b + TAS_G4c	25.39	21.01

Table S1: **Bayesian information criteria (BIC) and corrected Akaike information criteria (AICc) for logistic regression models predicting reversion from sequence features.**

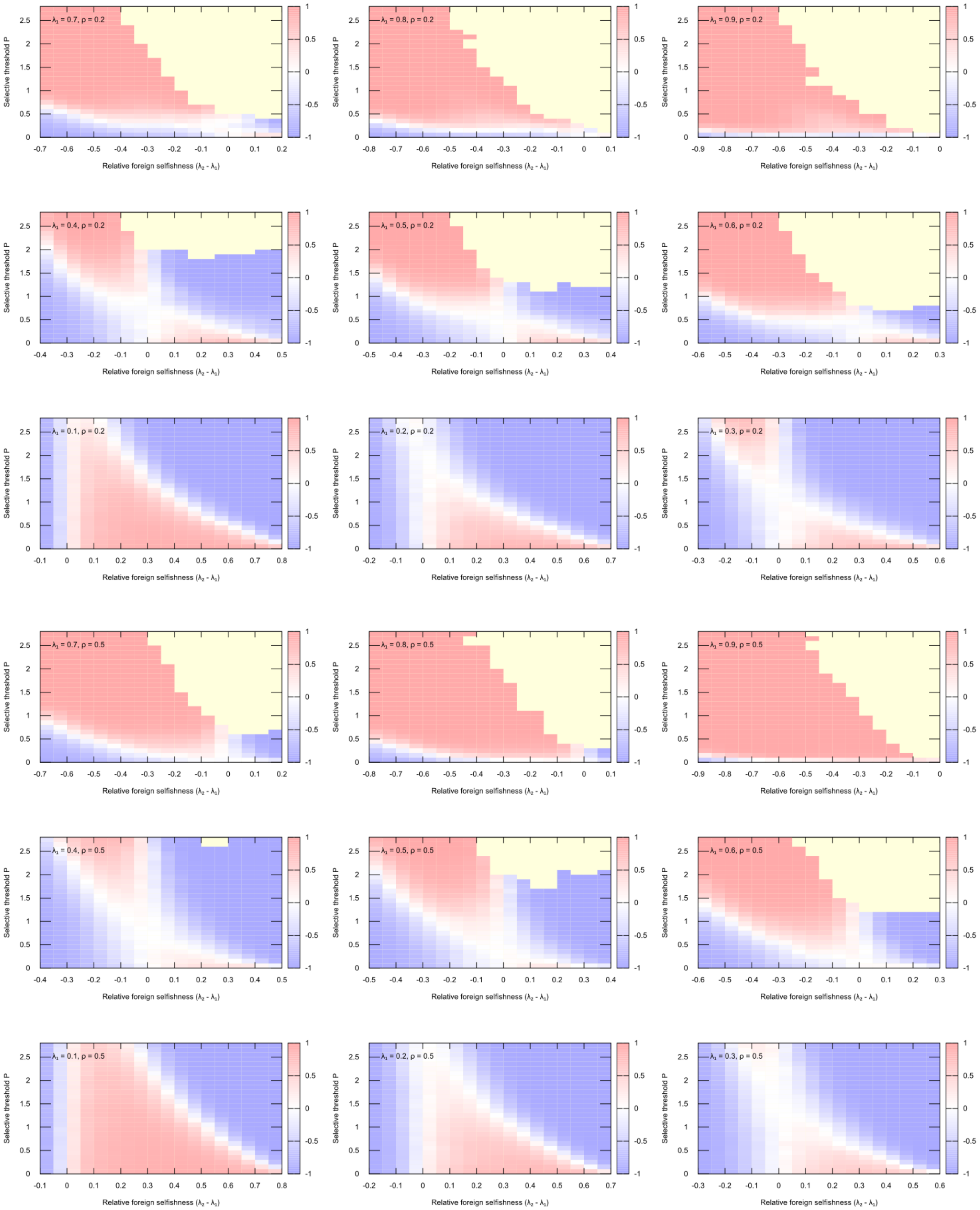

Figure S3: **RTS model with replication failure.** As in Supplementary Fig. S1, relative proliferation of the 'foreign' mtDNA type for different λ_1, with probability of replication failure ρ = 0.2 (top) and ρ = 0.5 (bottom). Yellow shaded regions (usually at the top right) denote regions of extinction (no mtDNAs are sufficiently unselfish to overcome selective pressure).

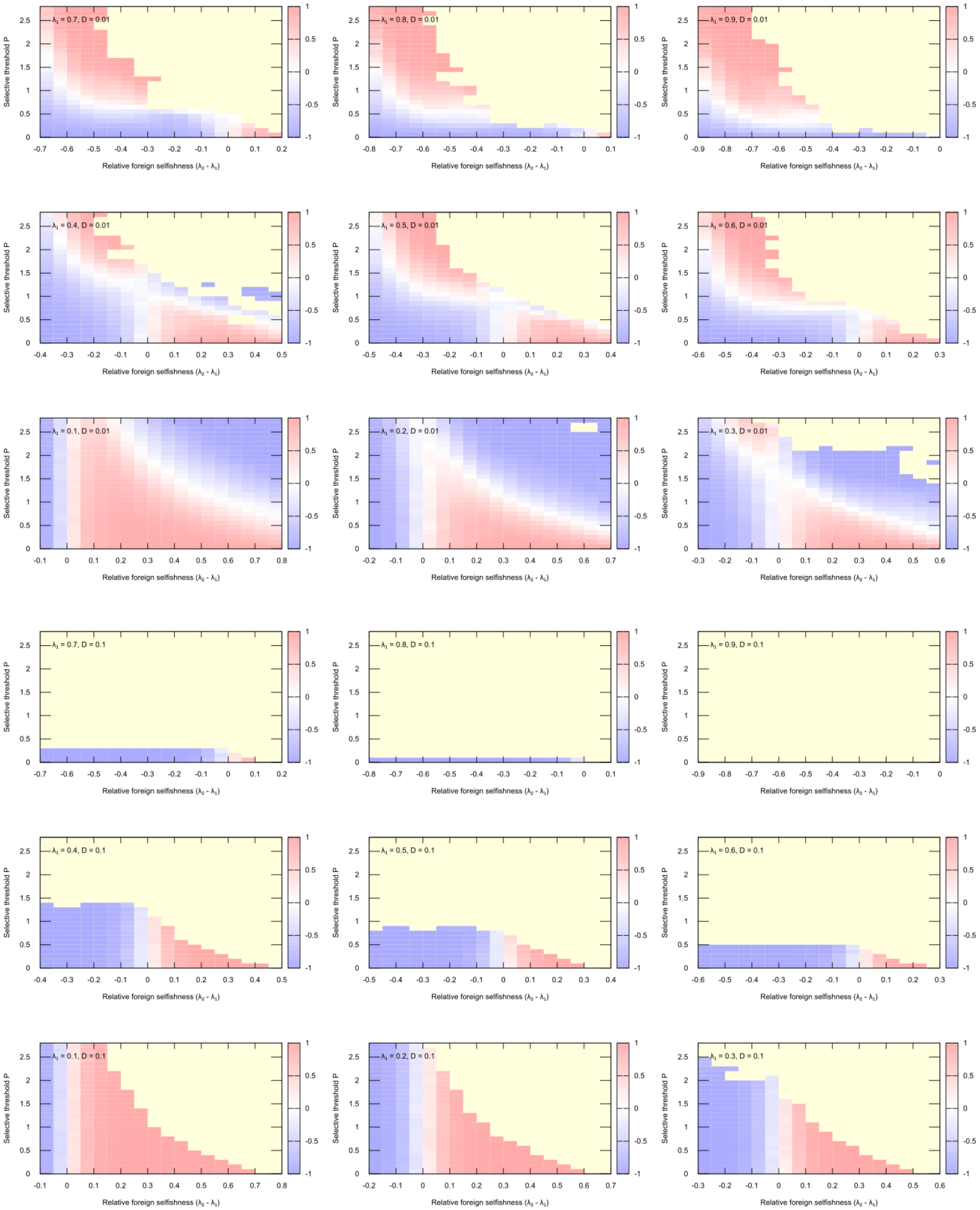

Figure S4: **RTS model with protein mixing.** As in Supplementary Fig. S1, relative proliferation of the 'foreign' mtDNA type for different λ_1, with protein sharing between mitochondrial elements with diffusion strength D = 0.01 (top) and D = 0.1 (bottom). Yellow shaded regions (usually at the top right) denote regions of extinction (no mtDNAs are sufficiently unselfish to overcome selective pressure).

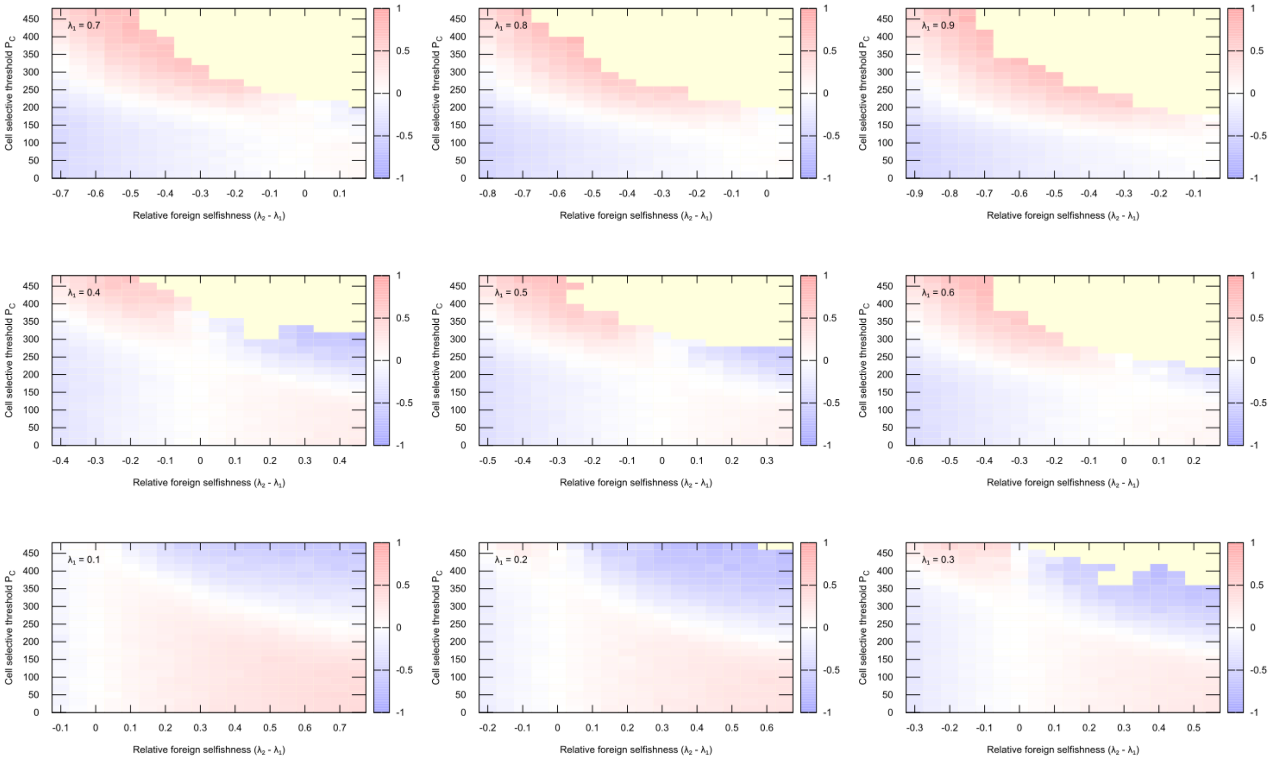

Figure S5: **Model with cell-level selection.** The relative proliferation of 'foreign' mtDNA (given by colour of the heatmap) in the cell-level model described in the Supplementary Information. The vertical axis now gives the cell-level selection threshold P_C. Yellow shaded regions (usually at the top right) denote regions of extinction (no mtDNAs are sufficiently unselfish to overcome selective pressure).

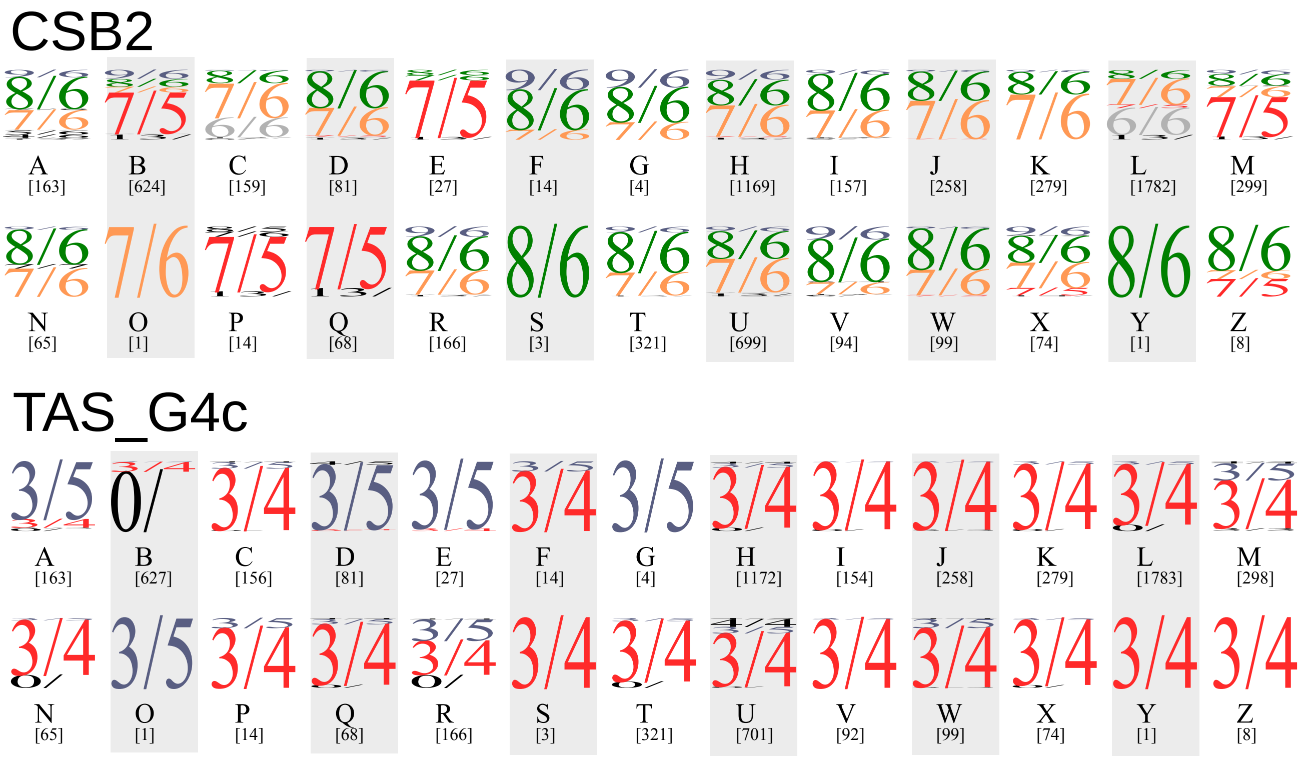

Figure S6: **Diversity in CSB2 and TAS G-quadruplex regions in human mtDNA.** Sequence characteristics of G-quadruplex regions in human mtDNA samples from NCBI, by haplotype. Number of accessions for each haplotype given in brackets. The height of a logo (*a*/*b*) gives the proportion of accessions in that haplotype that have *a* and *b* guanine residues flanking a central section in the corresponding G-quadruplex sequence.

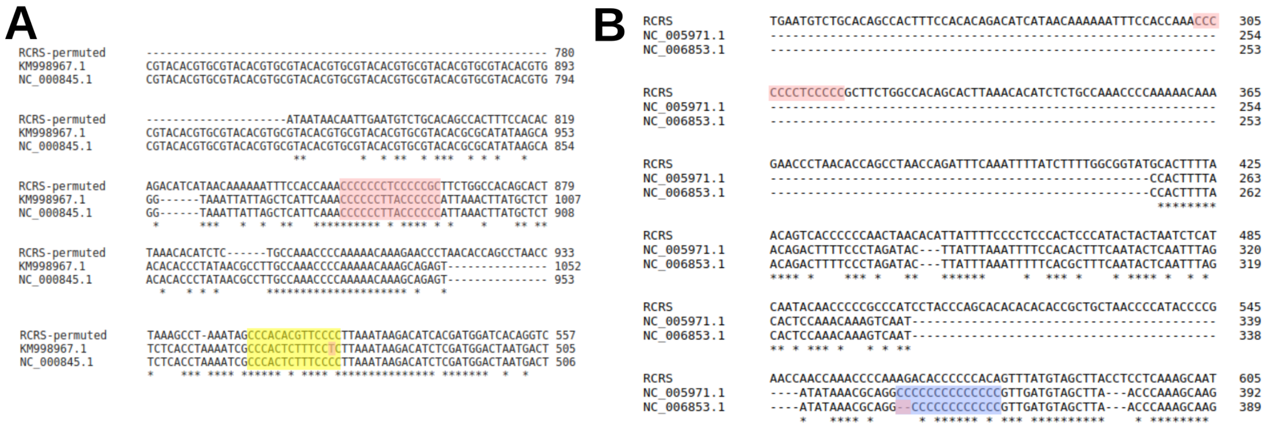

Figure S7: **Candidate G-quadruplex regions in livestock.** Clustal Omega [41] alignment of (A) pig and (B) cow mtDNA haplotypes, with human revised Cambridge reference sequence (RCRS) for reference. (A) Sections of Meishan (KM998967.1) and Landrace (NC_000845.1) *Sus scrofa* mitotypes, aligned with a permutation of the RCRS. No differences are observed between pigs in CSB2 (red), but a polymorphism impacts TAS_G4c (yellow). (B) *Bos indicus* (NC_005971.1), and *Bos taurus* (NC_006853.1) mtDNA. The cattle sequences do not contain the candidate G-quadruplex region aligning with human CSB2 (red) but do contain a nearby candidate region (blue), which differ in lengths of cystine bases.
